# Syphilis—Its Dangers and Results

**Published:** 1887-02

**Authors:** J. N. Martin

**Affiliations:** Lecturer on Oral Surgery and Pathology, Dental Dept., University of Michigan


THE DENTAL REGISTER.

VOL. XLI.] FEBRUARY, 1887. [No. 2.

### COMMUNICATONS.

### Syphilis—Its Dangers and Results.

BY J. N. MARTIN, M.D. LECTURER ON ORAL SURGERY AND PATHOLOGY, DENTAL DEPT., UNIVERSITY OF MICHIGAN.

In the catalogue of diseases there are few, if any, more to be dreaded than syphilis and especially by those who are cognizant of the terrible results of that disease.

There are many recorded cases of physicians and dentists being inoculated while treating patients who were infected with this terrible malady.

But few possess accurate knowledge on the dangers of inoculation and are aware of the terrible results that follow. To these dangers and results I wish to refer briefly, and in the near future will endeavor to make clear the signs and symptoms that characterize this disease, and the treatment that has proven most satisfactory.

*Causes.*—Syphilis comes from syphilitic virus, and from nothing else. There is a wide-spread opinion that one venereal disease "if allowed to run will run into another or others;" this is false; syphilis is always syphilis; gonorrhœa always gonorrhœa, and chancroid always chancroid; however, one may be infected with two or more of these diseases at the same time, and as a result have mixed signs and symptoms.

Syphilis is a constitutional disease with local manifestations, while the other two venereal diseases are local. Syphilis charges the whole system with its damnable influence before its local manifestations are noticeable.

How may this syphilitic virus which is in the blood and secretions of a syphilitic patient infect or inoculate one?

By sexual intercourse, vaccination, drinking cups, kissing, pipes, lead pencils, instruments used on patients with syphilis (coming in contact with blood and secretions); in fact, in any way that the blood or secretions of syphilitic patients are brought in contact with an abraded surface in one who has not had the disease, as in a wound, cut or scratch, fissure or crack, or an erosion.

It should always be kept in mind by dentists that secretions or blood from mouth of a patient suffering from syphilis may inoculate another if these secretions come in contact with an abraded surface.

I know of no more forcible illustration of the powers of this virus than the following, reported by the Government physician of Italy in 1861.

On June 7th, 1861, forty-six healthy children were vaccinated with lymph from one child, and on June the 12th, seventeen more with lymph from one of the forty-six. *Thirty-nine* of the *forty-six,* and seven of the seventeen took syphilis; total of forty-six out of sixty-three.

In October of same year, six of the forty-six had died, fourteen recovered, three in doubtful condition, and twenty-three dispersed through the country.

In addition, twenty of the women suckling the children were inoculated with syphilis by them, and through the mothers the disease reached several husbands and other children. It seems scarcely necessary to urge cleanliness on the part of dentists after such a record. Unfortunately this terrible disease is not confined to one rank of society.

As to the results of this disease: in addition to the effects on the general system by debility and paving the way for a long train of evils, it may attack every organ in the body; eyesight and hearing have been destroyed, taste lost, speech impaired or lost, brain rendered useless, lungs impaired, tongue, lips, and nose partially or wholly destroyed, bones attacked and bored full of holes, periostitis of most persistent form.

Effects on nervous system are numerous and varied—pains and aches everywhere, neuralgias of the most severe form, paralysis and especially paralysis of certain sets of muscles.

Again, teeth may crumble, hair drop out, and many other evil effects be produced by this disease

This disease is *dreadful* and *loathsome* to the one attacked but think of conveying it to husband or wife and causing it to be carried down the ages to generation after generation, making *many* homes miserable that might otherwise be full of blooming health and happiness, for this disease is certainly hereditary.

I firmly believe that the masses should be educated on this subject in order that they may know the dangers and shun them; for through ignorance many a pure chaste person has filled a syphilitic's grave.

